# Pseudothrombopénie non EDTA-dépendante: à propos d’un cas

**DOI:** 10.11604/pamj.2017.26.88.11097

**Published:** 2017-02-22

**Authors:** Hicham Titou, Youssef Jalal, Mohammed Boui

**Affiliations:** 1 Service de Dermatologie Vénérologie, Hôpital Militaire d’Instruction Mohamed V, Faculté de Médecine de Rabat, Maroc; 2 Service d’Orthopédie et de Traumatologie I, Hôpital Militaire d’Instruction Mohamed V, Rabat, Maroc

**Keywords:** Anticoagulant, héparine, plaquettes, pseudothrombopénie, Anticoagulant, heparin, platelets, pseudothrombocytopenia

## Abstract

La pseudothrombopénie est un phénomène rare de laboratoire, expliquée par une agglutination in vitro des plaquettes. L’examen au microscope du frottis du sang périphérique est un examen clé, pour confirmer le diagnostic et pour éviter toute décision clinique et thérapeutique inadaptée voir dangereuse. Sa survenue chez un patient, sous traitement par l’héparine, pose le problème de diagnostic différentiel avec la thrombopénie héparino-induite. Notre objective, à travers cette observation, est d’éviter toute confusion entre ce phénomène purement artéfactuel et la thrombopénie héparino-induite.

## Abstract (en)

Pseudothrombocytopenia is a rare phenomenon in laboratory explained by in vitro agglutination of platelets. Microscopic examination of the peripheral blood smear is of key importance to confirm the diagnosis and to avoid any inappropriate or dangerous clinical and therapeutic decision. Its occurrence in a patient, under treatment with heparin, raises the problem of differential diagnosis of heparin-induced thrombocytopenia. The aim of this study was to eliminate any confusion between this purely artefactual phenomenon and heparin-induced thrombocytopenia.

## Introduction

La pseudothrombopenie est une agglutination artéfactuelle de plaquettes in vitro, sans aucune signification clinique, de découverte souvent fortuite [1]. Pseudothrombopénie se produit habituellement dans le tube de prélèvement contenant de l´acide éthylène diamine tétra acétique (EDTA) ou un anticoagulant non-EDTA comme le citrate, l´oxalate et l´héparine [2]. Elle est expliquée par, la fixation in vitro des auto anticorps de type IgG sur les glycoprotéines GPII/III a de plaquettes activées [3]. La seule implication clinique, réside dans sa méconnaissance qui se traduit par un diagnostic erroné, des examens complémentaires inutiles et des traitements inappropriés [4]. En particulier, ce phénomène peut être confondu facilement avec une thrombopénie induite par l´héparine (TIH), chez les patients présentant une thrombopénie sous traitement par l´héparine [5]. TIH est une complication d´un traitement par l´héparine, expliquée par les anticorps antifacteur plaquettaire 4 (HPF-4), conduisant à l´activation des plaquettes et l’hypercoagulabilité [6]. L’objectif, à travers cette observation, est de mettre en lumière les pièges que peut présenter ce phénomène purement artéfactuel, et de faire le diagnostic différentiel avec une thrombopénie héparino-induite.

## Patient et observation

Un patient de 50 ans, était admis aux urgences, en juin 2015, pour polytraumatisme, suite à un accident de la voie publique. Il avait, comme antécédent, un ulcère gastrique depuis dix ans traité par inhibiteurs de la pompe à proton. Il ne décrivait jamais des épisodes de saignement anormaux. Il est suivi, en médecine de travail, depuis plusieurs années, sans hémogrammes anormaux. L’hémogramme à l’admission montrait un taux de plaquettes normal de 212.000/l, une hyperleucocytose à 39,8. 109/l et une hémoglobine à 13,3 g/dl. Les tests de coagulation de routine étaient normaux, montrant un taux de prothrombine à 90%, un temps de céphaline activé à 32 secondes et une fibrinogène à 3 g/l. Il recevait la ciprofloxacine associée à l’amoxicilline + acide clavulanique, pour une surinfection d’une fracture ouverte de la cuisse, par Pseudomonas stutzeri. Il bénéficiait d’une prophylaxie, par le sérum antitétanique et l’enoxaparine-Na également 4000 UI (en sous-cutanée), en raison de risque accru de thrombophlébite, à cause de l´immobilité et de polyfractures. La recherche de plasmodium sur goutte épaisse était négative. Egalement, les sérologies VIH, HVB et HVC étaient négatives. Après parage et soins locaux, un traitement par ostéosynthèse et fixateurs externes était programmé 2 semaines après. Au bilan pré-anesthésique, l’hémogramme sur tube à EDTA, montrait les résultats suivants: un taux de plaquettes à 84.000/l, avec la présence d’une alarme d’agrégats plaquettaires indiquant un volume plaquettaire moyen de 11,2 fl, des globules blanc à 7,8 109/l, l’hémoglobine à 9,4 g/dl et l’hématocrite à 28,3%. Le contrôle sur lame, par le microscope, confirmait la présence de nombreux agrégats plaquettaires. L’hémogramme contrôlé sur tube citraté, montrait les résultats suivants : un taux de plaquette à 65.000/l, avec alarme d’agrégats plaquettaires, indiquant un volume plaquettaire moyen à 11,3 fl. Le contrôle sur lame trouvait également des agrégats plaquettaires. Un prélèvement sur tube EDTA préchauffé à 37°c, et maintenue à cette température jusqu’au moment de l’analyse, montrait des résultats semblables. Tous ces prélèvements ont été analysés par l’automate de type Coulter LH 750. Le diagnostic d’une PTP non EDTA-dépendante était retenu. Le traitement par l’héparine était poursuivi, avec des suites post opératoires normaux. 5 jours après l’opération, l’hémogramme montrait toujours une pseudothrombopénie non EDTA-dépendante sous héparinothérapie prophylaxique.

## Discussion

La thrombopénie peut être due à une augmentation de la destruction des plaquettes, ou à une diminution de la production des plaquettes. Elle peut aussi être causée par, la fixation in vitro des anticorps de type agglutinine sur les récepteurs GPIIb/III a des plaquettes activées déclenchant une agrégation plaquettaire, sans aucun signe clinique de saignement, qui est appelée Pseudothrombopénie [7]. Schrezenmeier et collègues ont trouvés que non seulement l´EDTA peut entrainer une agglutination in vitro, mais également l´oxalate de sodium et le citrate de sodium [8]. En cas de suspicion d’une pseudothrombopénie liée à l’EDTA, un anticoagulant différent comme le citrate, l’oxalate ou l’héparine doit être utilisé pour le comptage des plaquettes [7]. Le citrate de sodium est un chélateur d’ions de calcium, comme l’EDTA, mais il est rarement la cause d’agrégat plaquettaire [6]. L’héparine peut entrainer un agrégat plaquettaire, mais il ne chélate pas les ions de calcium [6]. Dans notre cas, une pseudothrombopénie non EDTA-dépendante a été confirmée, par frottis du sang périphérique sur tube à EDTA et sur tube citraté, en respectant une température ambiante à37c. C’est une entité clinique rare. Bizzaro a rapporté que 20% d´une large série de patients, atteints de pseudothombopenie, développent une agglutination in vitro des plaquettes non seulement avec l´EDTA, mais également avec le citrate [9].

**Figure 1:**
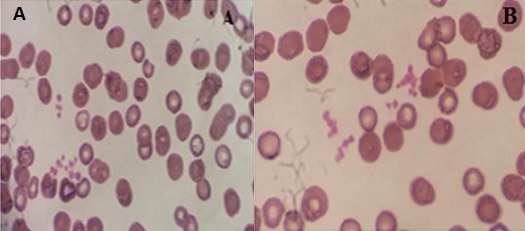
Frottis du sang périphérique montrant des agrégats plaquettaires sur tube à EDATA(A) et sur tube citraté (B) (May–Grünwald–Giemsa [MGG] 100×)

Pseudothrobopénie a été documentée chez des patients, affectés par une grande variabilité de pathologies, comme les maladies auto-immunes, les maladies inflammatoires chroniques, les infections virales et bactériennes, les syndromes métaboliques et les sujets sains. Ces différentes situations cliniques, confirment que la pseudothrombopénie n’est pas causée ou associée à une pathologie spécifique ou à l’utilisation des médicaments particuliers [4]. Dans la littérature, des cas de pseudothrombopénie ont été décrites, en rapports avec des médicaments couramment utilisés en milieu hospitalier, comme l’insuline et l’héparine [6]. Dans notre cas, le patient a présenté à l’admission une numération plaquettaire normale. Un traitement, par héparine de bas poids moléculaire, a été administré à dose prophylaxique, vu le risque de thrombophlébite à cause de l’immobilité et de polyfractures. Au bilan anesthésique 2 semaines après, l’hémogramme montrait une thrombopénie profonde. Le diagnostic d’TIH a été évoqué, avec discussion d’arrêt de l’héparine et une éventuelle transfusion des plaquettes. Cependant, la réalisation d’un frottis du sang périphérique a objectivé des agrégats plaquettaires, avec un taux des plaquettes par comptage manuel à 223.480/l, confirmant ainsi le diagnostic de pseudothrombopénie (Figure 1). Le patient n’a pas été transfusé, l’héparinothérapie a été poursuivie et aucune complication hémorragique ou thrombotique n’a été détectée.

TIH doit être évoqué habituellement, chez les patients qui développent une thrombopénie, dans les 5 jours qui suivent le début d’un traitement par l’héparine, lorsque les autres causes de thrombopénie sont exclues. Exceptionnellement, l’TIH peut survenir quelques heures, après l’administration de l’héparine, lorsque le patient a été traité par l’héparine dans les 120 jours qui précèdent. Dans notre cas, TIH était exclue devant l’absence d’une thrombopénie réelle, l’absence d’antécédent de traitement par l’héparine dans les derniers 120 jour et l’absence de signes cliniques de saignement ou de thrombose.

Un autre aspect intéressant, est celui des conséquences possibles de la transfusion de sang, provenant d´un donneur avec pseudothrombopénie. Sweeney et al [10], ne trouvent pas de problèmes chez les transfusés, y compris l’absence d’apparition de pseudothrombopénie chez les receveurs du sang, ce qui indique que le sang des sujets sains avec pseudothrombopénie peut être utilisé en thérapie transfusionnelle.

Aucun cas familial n’a été documenté, mais un cas de pseudothrombopénie congénitale transitoire, dû à la transmission transplacentaire des anticorps de type IgG de la mère au fœtus a été décrit [11].

## Conclusion

Ce cas a démontré qu’une thrombopénie préopératoire peut être, dans des rares occasions, le résultat d’un artéfact de laboratoire. Un examen au microscope du frottis de sang périphérique, permet le diagnostic différentiel entre pseudothrombopénie et thrombopénie réelle. La méconnaissance d’une pseudothrombopenie peut entrainer des investigations complémentaires inutiles, une interruption d’intervention chirurgicale et des décisions thérapeutiques inefficaces et parfois dangereuses. Chez les patients candidats à la chirurgie, et qui présentent une thrombopénie, une analyse critique est recommandée si absence d’histoire clinique de saignement, avec une grande variabilité de la numération plaquettaire. Lorsque la thrombopénie est survenue chez un patient sous traitement par l’héparine, une pseudothrombopénie doit être exclue avant de poser le diagnostic d’TIH.
